# The Phenotypic Profile Associated With the *FMR1* Premutation in Women: An Investigation of Clinical-Behavioral, Social-Cognitive, and Executive Abilities

**DOI:** 10.3389/fpsyt.2021.718485

**Published:** 2021-08-06

**Authors:** Nell Maltman, Janna Guilfoyle, Kritika Nayar, Gary E. Martin, Molly Winston, Joseph C. Y. Lau, Lauren Bush, Shivani Patel, Michelle Lee, John Sideris, Deborah A. Hall, Lili Zhou, Kevin Sharp, Elizabeth Berry-Kravis, Molly Losh

**Affiliations:** ^1^Roxelyn and Richard Pepper Department of Communication Sciences and Disorders, Northwestern University, Evanston, IL, United States; ^2^Waisman Center, University of Wisconsin-Madison, Madison, WI, United States; ^3^Department of Communication Sciences and Disorders, St. John's University, Staten Island, NY, United States; ^4^Chan Division of Occupational Science and Occupational Therapy, University of Southern California, Los Angeles, CA, United States; ^5^Department of Neurological Sciences, Rush University, Chicago, IL, United States; ^6^Rush University Medical Center, Chicago, IL, United States

**Keywords:** *FMR1* premutation, broad autism phenotype, latent profile analysis, executive function, social cognition

## Abstract

The *FMR1* gene in its premutation (PM) state has been linked to a range of clinical and subclinical phenotypes among *FMR1* PM carriers, including some subclinical traits associated with autism spectrum disorder (ASD). This study attempted to further characterize the phenotypic profile associated with the *FMR1* PM by studying a battery of assessments examining clinical-behavioral traits, social-cognitive, and executive abilities in women carrying the *FMR1* PM, and associations with *FMR1*-related variability. Participants included 152 female *FMR1* PM carriers and 75 female controls who were similar in age and IQ, and screened for neuromotor impairments or signs of fragile X-associated tremor/ataxia syndrome. The phenotypic battery included assessments of ASD-related personality and language (i.e., pragmatic) traits, symptoms of anxiety and depression, four different social-cognitive tasks that tapped the ability to read internal states and emotions based on different cues (e.g., facial expressions, biological motion, and complex social scenes), and a measure of executive function. Results revealed a complex phenotypic profile among the PM carrier group, where subtle differences were observed in pragmatic language, executive function, and social-cognitive tasks that involved evaluating basic emotions and trustworthiness. The PM carrier group also showed elevated rates of ASD-related personality traits. In contrast, PM carriers performed similarly to controls on social-cognitive tasks that involved reliance on faces and biological motion. The PM group did not differ from controls on self-reported depression or anxiety symptoms. Using latent profile analysis, we observed three distinct subgroups of PM carriers who varied considerably in their performance across tasks. Among PM carriers, CGG repeat length was a significant predictor of pragmatic language violations. Results suggest a nuanced phenotypic profile characterized by subtle differences in select clinical-behavioral, social-cognitive, and executive abilities associated with the *FMR1* PM in women.

## Introduction

The *FMR1* gene plays a critical role in the expression of a range of clinical phenotypes, including both neurodevelopmental and neurodegenerative disorders. Located in the *5*′ *untranslated region (5*′ *UTR)* on the long arm of the X chromosome, *FMR1* encodes the Fragile X Mental Retardation Protein (FMRP), which is highly expressed in the brain and plays a role in synaptic plasticity (1–4). A full mutation of the *FMR1* gene (>200 cytosine-guanine-guanine [CGG] trinucleotide repeats) causes methylation and subsequent silencing of the gene, inhibiting production of FMRP and causing fragile X syndrome (FXS), a rare condition (~1 in 4,000 males and ~1 in 8,000 females) that is the most common inherited cause of intellectual disability and monogenic cause of autism spectrum disorder (ASD) (5–7). The *FMR1* premutation (PM; 55–200 CGG repeats), occurs in roughly 1 in 150–250 women, and is less prevalent in men (~1 in 430–460) (8–12). Though the PM was once believed to have limited phenotypic expression, a number of clinical and subclinical phenotypes have since been identified, including PM-specific disorders (i.e., fragile X-associated primary ovarian insufficiency [FXPOI], fragile X-associated tremor/ataxia syndrome [FXTAS], and fragile X-associated neuropsychiatric disorders [FXAND]) (4, 13, 14). *FMR1*-related molecular genetic variation has been associated with these phenotypes, including CGG repeat length, such as mid-range vulnerability (90–110 repeats), toxic gain-of-function production of mRNA, RAN translation, and FMRP variation (15–23). As such, detailed phenotypic characterization of the PM is important from a public health perspective, with potential to connect complex human traits to known genetic variation.

An important body of work has described a number of clinical-behavioral traits among carriers of the *FMR1* PM, including ASD and subclinical ASD-related traits (24–27), anxiety and depression (28–33), and differences in social cognition and executive function (EF) (34–38). Because most prior work has examined these phenotypes in separate study samples, a key remaining question concerns whether such phenotypes co-occur, together constituting a phenotypic profile associated with the *FMR1* PM. Examining the co-occurrence of key phenotypes within individuals can also help to address whether such features may interrelate causally. It could be, for instance, that personality traits previously reported in PM groups reflect underlying differences in social cognition or EF. This study attempted to build on prior work to address these gaps by studying a range of clinical-behavioral, social-cognitive, and executive phenotypes associated with the *FMR1* PM within a relatively large sample of female PM carriers and controls.

Consistent with the strong phenotypic overlap observed between FXS and ASD (where most individuals with FXS exhibit at least some ASD symptoms, and many meet full diagnostic criteria for ASD) (24, 39–42), a number of studies have documented elevated rates of ASD among PM carriers, particularly males (~14%) (24, 25, 43). Subclinical ASD traits have also been noted in the PM more generally, including personality styles such as social reticence (28) and rigid and perfectionistic traits (27, 34). Collectively known as the broad autism phenotype (BAP), this constellation of subclinical personality and language traits mirror the central features of ASD and are thought to index genetic liability to the disorder as they are observed at higher rates among first degree relatives of individuals with ASD relative to the general population (44–46) and associated with increased polygenic burden for ASD (47).

Losh et al. (27) evaluated personality and pragmatic language features of the BAP in PM carriers. Using direct assessment measures, including the Modified Personality Assessment Scale (MPAS) (48) and Pragmatic Rating Scale (PRS) (49), they found similar profiles of BAP personality traits and increased pragmatic language violations in PM carriers and mothers of individuals with ASD compared to controls. Further, within-family associations were detected in the PM group, showing that children with FXS whose mothers exhibited BAP traits had more severe ASD symptoms. Such co-segregation of ASD-related phenotypes within a subgroup of families is intriguing, particularly when considering known interactions between a number of ASD risk genes and the *FMR1* gene that might underlie these phenotypes (50, 51).

Beyond ASD-related risk, female PM carriers may display elevated symptoms of anxiety and depression, though rates vary considerably between studies (30, 32, 33, 52, 53). For instance, whereas Jiraanont et al. (53) reported higher diagnostic rates of depression (50%) and anxiety (33%) compared to controls (8.3 vs. 4.2%, respectively), Gossett et al. (52) reported no significant differences, but noted that the majority of the control group (~54%) had clinically elevated symptoms. Studies including younger PM carriers and adults without children have also reported elevated mood and anxiety disorders, suggesting that these symptoms may arise early on and are not merely related to the stress of parenting a child with FXS (25, 32). Interestingly, there is some evidence to suggest that mood and anxiety symptoms may co-occur with other phenotypes, such as FXPOI and EF deficits (e.g., working memory), among subgroups of PM carriers (54, 55), underscoring the importance of examining how these symptoms may co-occur with other phenotypes, such as social-cognitive deficits and ASD-related features.

Subtle differences in social cognition (i.e., understanding mental states and feelings essential for supporting fluent interpersonal interactions) have also been observed among PM carriers. Studies of male PM carriers reported lower social-cognitive performance, including decreased theory of mind (34, 56) and reduced social reward processing, which related to lower FMRP (37). Male PM carriers have been reported to exhibit reduced neural activation in the amygdala compared to controls when viewing faces with fearful expressions (35). Far less is known about the social-cognitive profiles of females and potential biological correlates. Klusek et al. (57) found that, compared to controls, female PM carriers displayed reduced visual attention to others' direct gaze, which can impact the ability to interpret others' intent. These results were hypothesized to reflect difficulty with interpreting ambiguous social information or with recognizing direct gaze as an important social cue.

Even subtly impaired social-cognitive abilities have been associated with increased pragmatic language violations in ASD and FXS and the *FMR1* PM (58–62). For example, subgroups of parents of individuals with ASD who displayed reduced social-cognitive skills tended to show elevated pragmatic language violations during semi-structured conversation (60). In FXS, increased pragmatic language deficits have been reported to cosegregate with more severe impairments in social cognition (62), and some evidence suggests that differences in social cognition among PM carriers may also relate to pragmatic language. In a study of visual attention, Winston et al. (63) reported atypical visual scanning patterns of faces among female PM carriers, but found that these differences were associated with *better* social cognition and pragmatic language, perhaps suggesting that some female PM carriers may employ alternative strategies for deducing meaningful information in social exchanges. Such findings suggest that subgroups of female PM carriers may exhibit social-cognitive or attentional patterns that facilitate pragmatic language, whereas for others, these domains may not be related. Consistent with this possibility, Winston et al. (64) identified a subgroup of PM carriers who demonstrated social viewing patterns characteristic of those found among parents of individuals with ASD and greater co-occurring pragmatic difficulties, and also had children with more severe ASD symptoms.

Numerous studies have also identified EF differences among female PM carriers without FXTAS (36, 65–67), including differences in working memory across both visual and verbal modalities (68, 69), inhibition (23, 54, 70), and attention (71). PM carriers also exhibit differences in language domains that are thought to reflect underlying EF difficulties, such as verbal disfluencies (72–74). Additionally, a recent study reported inefficient language processing and eye-voice coordination on a task of rapid naming of familiar objects; difficulties were particularly evident during the latter portions of the task when executive demands have been shown to be the greatest (22). The overlap between these language tasks and EF could suggest that aspects of the PM phenotype, such as subtle differences in pragmatic language, could cluster together with EF difficulties. For instance, Kraan et al. (54) found that EF deficits co-occurred with neuropsychiatric symptoms (such as depression and anxiety) in female PM carriers, though far less is known about the co-occurrence of executive dysfunction and other clinical-behavioral or cognitive correlates.

The present study aimed to further characterize the phenotypic spectrum associated with the *FMR1* PM by examining performance across a battery of clinical-behavioral, social-cognitive, and EF tasks in females with the *FMR1* PM in comparison to controls. Further, we utilized latent profile analyses to examine whether homogenous phenotypic subgroups within the PM might be identified based on distinct constellations of these phenotypes. Finally, following prior evidence that *FMR1*-related molecular-genetic variation is often associated with clinical-behavioral and cognitive phenotypes in the PM (21, 23, 30, 33, 37, 56, 75–77), we examined associations between the phenotypic battery and CGG repeat length and FMRP.

## Methods

### Participants

Participants included 152 female *FMR1* PM carriers and 75 adult female controls. Only females were included in this study to control for biological sex, and to ensure feasible ascertainment. All participants were native English speakers because of the language-based nature of many of the tasks. PM carriers were recruited from genetic clinics, physicians' offices, advocacy groups, and the Research Participant Registry Core of the Carolina Institute for Developmental Disabilities at the University of North Carolina at Chapel Hill. PM status was confirmed by genetic testing (direct or confirmation by prior medical records). Controls were recruited through community resources (e.g., schools and child care centers, local community events) and word of mouth, as well as through the Communication Research Registry at Northwestern University. Controls were recruited as part of larger family genetics studies of ASD/BAP and *FMR1*-related conditions, and were therefore screened for personal or family history of FXS, ASD, and genetically-based conditions associated with ASD. Table 1 summarizes participant characteristics. Groups did not differ significantly in age (*p* = 0.233), or Full-Scale IQ (FSIQ; *p* = 0.250). In the PM sample, 135 participants had at least one child with FXS. Nine participants were mothers of children without FXS, and 16 were not mothers.

**Table 1:** Participant characteristics.

	**Premutation carriers**	**Female controls**
	***M* (*SD*)**	***M* (*SD*)**
*N*	152	75
Chronological age	44.43 (8.76)	42.50 (9.21)
IQ	112.90 (9.78)	114.67 (11.20)
VIQ	110.69 (10.59)	110.29 (12.20)
PIQ	**111.97 (9.58)**	**113.45 (13.52)**

*VIQ, Verbal IQ; PIQ, Performance IQ*.

*Participants did not significantly differ on age, IQ, or VIQ (ps > 0.230), but did differ on PIQ (p = 0.039; in bold)*.

Potential participants were asked to report any prior diagnosis of FXTAS or Parkinsonism, and were excluded if they endorsed such symptoms. Additionally, participants completed a reduced set of screening questions from the FXTAS Rating Scale (78, 79), assessing action or postural tremor, standing capacities, tandem gait, and handwriting-related items. Four individuals were excluded for rating positive on one or both of these indices.

All study procedures were approved by the Institutional Review Boards of participating universities. All participants provided informed consent to participate.

### Clinical-Behavioral Measures

#### Assessment of the BAP

##### Modified Personality Assessment Schedule

The Modified Personality Assessment Schedule (MPAS) (48) was used to assess three core personality traits associated with the BAP (i.e., social aloofness, rigidity, and untactfulness) among the PM carrier group. This instrument has been used extensively in family studies of ASD [e.g., (44, 45)], and consists of a direct-assessment clinical interview designed to elicit examples regarding the endorsement of each personality trait. Traits are rated based on concrete behavioral examples using a 3-point scale (0 = absent, 1 = partially present, and 2 = present). Given that the BAP occurs at relatively low rates in individuals without a family history of ASD (46, 80), and the overarching goal of assessing how BAP personality traits might relate to other phenotypes among PM carriers, controls were not administered the MPAS.

Each interview was coded by two independent raters who were trained to at least 80% reliability, and were blind to group status, with final scores determined through consensus. Individuals were rated BAP (+) for social features if they had a score of 2 on either social aloofness or untactfulness, and BAP (+) for rigid features if they had a score of 2 in the rigid domain. Finally, individuals were rated BAP (–) if they scored either a 0 or 1 on all domains. Average reliability prior to consensus coding was 76.28%.

##### Pragmatic Rating Scale

Twenty-minute semi-structured conversations were conducted between examiners and participants concerning their “life history” [for detailed description, see (27)]. Participants were asked about a series of topics that pertained to early childhood and friendships, current employment, hobbies, and romantic relationships. To elicit specific pragmatic behaviors during the conversation, such as reciprocity and the ability to clarify a message, examiners were trained to periodically offer related personal information and feign confusion. Conversations were coded from video by two trained research assistants, who were blind to participant family diagnosis, using the Pragmatic Rating Scale (PRS) (49). The PRS captures pragmatic language features of the BAP, and assesses 26 different pragmatic skills (e.g., providing adequate detail and background information), which are rated on a three-point scale from 0 (not present), 1 (somewhat present), to 2 (definitely present). In addition to a total number of pragmatic language violations, scores on three factor scores [dominant, withdrawn, and suprasegmental factors; see (27)] were also examined. All files were consensus coded for a best estimate rating used in analyses. Reliability prior to consensus coding was 84.07%.

#### Mood and Anxiety

##### Beck Depression Inventory-II

Depression symptoms were assessed using the Beck Depression Inventory-II (BDI-II) (81). The BDI-II is a 21-item measure of depressive symptoms on a 4-point scale, and has been normed in both typical and clinical populations. It follows the Diagnostic and Statistical Manual of Mental Disorders-Fourth Edition (DSM-IV) criteria for depression and assesses the presence and severity of depression with high reliability (Coefficient Alpha = 0.92). Scores on this measure range from 0 to 63; scores falling in the 0–13 range suggest the presence of minimal depressive symptoms, 14–19 indicate mild, 20–28 moderate, and 29–63 severe.

##### State-Trait Anxiety Inventory

The State-Trait Anxiety Inventory (STAI) (82) assessed anxiety symptoms. Used extensively in psychiatric research and practice, the STAI consists of two 20-item questionnaires that evaluate current (i.e., state) and more persistent (i.e., trait) symptoms of anxiety. The STAI provides a continuous measure of anxiety, with a range for each subtest of 20–80, with higher scores indicating greater anxiety (83, 84). Clinically significant anxiety has been suggested for scores at or above 39 (83, 84). Standard scores were used in all analyses.

##### Mini-International Neuropsychiatric Interview

The Mini-International Neuropsychiatric Interview (MINI) (85) is a well-validated and widely used structured psychiatric diagnostic interview instrument to evaluate current and past depressive episodes along DSM-IV criteria. This measure is intended as a tool for dichotomous categorization (i.e., yes/no) of psychiatric symptoms of depression and anxiety. Two-sided Fisher's exact tests were used to compare rates of symptoms across groups.

### Social Cognition

#### Reading the Mind From the Eyes Task

The Eyes Task (86) was used to index the ability to infer psychological states from viewing the eye region of the face. Participants were shown 36 different images of eyes expressing different psychological states and were asked to select a corresponding term from an array of four words. Performance was measured as the proportion of correct responses.

#### Trustworthiness of Faces Task

The Trustworthiness of Faces Task (87, 88) assessed the ability to use facial expressions to infer the social attribute of trustworthiness. Participants were asked to rate the trustworthiness of a series of 42 faces, which varied in gender, expression, and gaze. Ratings were made on a seven-point scale (−3 to +3), with negative scores denoting less trustworthiness, a score of “0” denoting neutral trustworthiness, and positive scores indicating greater trustworthiness. Faces were categorized into “negatively valenced” and “positively valenced” based on valence ratings from the original control group (87).

#### The Movie Stills Task

The Movie Stills Task (89) measured the extent to which individuals use facial information to infer the emotional content of a scene. Participants were asked to determine the emotional state (happy, sad, afraid, angry, surprised, disgusted, or neutral) from a series of 16 movie stills. The first trial obscured the faces of the characters in the image, and the second trial presented the image with the faces intact. Following prior work (89), scores were derived from the control participants (*n* = 49). For each of the 16 images with faces, the proportion of controls who selected a given emotion was first calculated to determine the distribution of responses. Scores were then weighted based on the distribution of responses, and parametrically transformed to give partial credit to alternative responses. For instance, if on a given image, 50% of the controls chose angry, 40% chose afraid, and 10% chose neutral, angry was given a score of 1, afraid was weighted as 0.8, and a neutral response was weighted as 0.2, whereas all other responses received no credit. For each image, the emotion that was the most often chosen by the controls was determined as the target emotion. Scores were then averaged for each emotion condition.

#### Point Light Tasks

The ability to recognize emotions through biological motion was assessed using a task where participants viewed light emitting diodes affixed to an actor's body, as the figure moved through a black space in different manners (90). Participants completed two versions of this task—one in which they judged basic emotions (e.g., happy, angry) and another where they judged the trustworthiness conveyed by the body movements. As with the Movie Stills task, each emotion was given a weighted score based on the proportion of controls who selected each emotion per trial. The weighted scores were used to calculate emotional accuracy.

During the second version of the task, participants were asked to rate the trustworthiness of each display based on its pattern of movement. Trustworthiness was rated on a scale of 1–5, with 1 representing the most trustworthy and 5 representing the least trustworthy. For analyses and interpretation of this version, images were divided into “lower” and “higher” trustworthiness based on original control ratings. Raw ratings of trustworthiness were used in analyses.

### Executive Function

#### Behavior Rating Inventory of Executive Function—Adult Version

Participants completed the Behavior Rating Inventory of Executive Function-Adult Version (BRIEF-A) (91), a 75-item self-report questionnaire that assesses multiple domains of executive functioning. Participants had the option to complete this self-report questionnaire in the lab, or remotely *via* an online link to the questionnaire. Each item was rated on a three-point Likert scale (i.e., never, sometimes, often), indicating the extent to which a behavior occurred over the past 6 months. Raw scores were converted into standardized t-scores across nine domains: Inhibition, Shift, Emotional Control, Self-Monitor, Initiate, Working Memory, Plan/Organize, Task Monitor, and Organization of Materials. Higher scores indicate poorer executive functioning abilities. Scores from each domain yield an overall Global Executive Composite (GEC) score, and two composite scores, Behavioral Regulation Index (BRI) and Metacognitive Index (MI). *T*-scores 65 or greater indicate clinically significant deficits in executive functioning.

### *FMR1* Molecular-Genetic Variation

Polymerase chain reaction and Southern blot techniques were used to confirm PM status and determine CGG repeat length. FMRP was assayed in lymphocytes isolated from blood, using a Luminex Assay to reliably quantify levels of FMRP (92). Activation ratio (AR) measures the proportion of cells carrying the normal allele on the active X chromosome (78), and was determined by the ratio of the intensity of the normal *FMR1* unmethylated band divided by the sum of the intensities of the normal unmethylated and methylated bands (93). Table 2 presents descriptive information regarding *FMR1*-related variation.

**Table 2:** *FMR1* characteristics in PM carriers.

	***M* (*SD*)**	**Range**
CGG repeat length	89.68 (16.23)	59–126
Quantitative FMRP	0.02 (0.01)	0.00–0.04
Activation ratio	0.49 (0.23)	0.00–0.95

*CGG, Cytosine-Guanine-Guanine; FMRP, fragile X mental retardation protein (pg/ug). Activation ratio reflects the percentage of cells for which the active X chromosome contains an unaffected FMR1 gene*.

### Data Analysis

All variables were examined for normality of distribution. General linear models (i.e., analyses of variance, ANOVAs) were used to assess group differences across clinical-behavioral, social-cognitive, and EF measures. Effect sizes for correlational analyses were interpreted as small (0.20), medium (0.50), and large (0.80) (94). Effect sizes for ANOVAs are reflected as partial eta squared ($\eta_{\text{p}}^{2}$), and were interpreted as small (0.01), medium (0.06), and large (0.14) (94). Bonferroni corrections were applied to adjust for multiple comparisons. Because groups did not differ significantly on age or IQ (*p* > 0.153), and age and IQ were generally not associated with variables of interest, we did not control for these variables in analyses.

To examine whether there may be subgroups who display specific patterns of performance across tasks, we conducted latent profile analyses (LPA) in the PM group. LPA serves to identify latent subpopulations having different configural profiles based on variables hypothesized to comprise meaningful phenotypes (95). A total of 14 variables reflecting performance across measures were included as numerical indicators in the LPA (see Table 3). Given numerical scale differences between measures, all variables were *z*-scored to improve interpretability.

**Table 3:** Measures included as numerical indicators in latent profile analysis (LPA).

**Domain**	**Measures**	**Variables**
Personality Features of the BAP	Modified Personality Assessment Schedule—Revised (MPAS)	1. MPAS-Social (social aloofness + untactful) 2. MPAS-Rigid
Pragmatic Language Features of the BAP	Pragmatic Rating Scale (PRS)	1. PRS-Dominating Conversation factor score 2. PRS-Withdrawn factor score 3. PRS-Suprasegmentals factor score
Executive Functioning	Brief Rating Inventory of Executive Function (BRIEF)	1. BRIEF—Global Executive Composite *T*-score
Mood and Anxiety	Beck Depression Inventory (BDI) The State-Trait Anxiety Inventory (STAI)	1. BDI total score 2. State Anxiety subscale total score 3. Trait Anxiety subscale total score
Social Cognition	Reading the Mind in the Eyes Task Trustworthiness of Faces Task Movie Stills Task Point Light Basic Task	1. Eyes Task percent correct 2. Trustworthiness mean ratings 3. Movie skills with faces mean emotion ratings 4. Movie Stills without faces mean emotion ratings 5. Point Light mean emotion ratings

*All variables were z-scored prior to inclusion in the LPA. BAP, Broad autism phenotype*.

Using an iterative process, we evaluated LPA solutions ranging from one to six potential PM profiles using the following fit measures to determine the best solution: the Akaike Information Criterion (AIC), the Bayesian Information Criterion (BIC), Entropy, a Bootstrap Likelihood Ratio Test (BLRT), and a Sattora–Bentler Scaled likelihood ratio chi-square difference test (TRd) (95–98). We also considered the theoretical interpretability of the profiles and profile size (for technical details on LPA, see Supplementary Materials). Profile subgroups were examined in follow-up analyses to evaluate any meaningful differences in age, IQ, *FMR1*-related genetic variation (i.e., CGG repeat length, AR, and quantitative FMRP), maternal status, or severity of ASD symptoms in their children (measured in oldest child in multiplex families using the ADOS-2) (99).

Associations between clinical-behavioral and cognitive phenotypes with molecular-genetic variables (i.e., *FMR1* CGG repeat length, FMRP) were conducted in the PM group only using Pearson correlations. Subsequently, linear regression models were applied to examine the extent to which molecular-genetic variables might predict phenotypic outcomes. For regression models that included CGG repeat length, separate models were conducted using linear and curvilinear CGG terms (i.e., CGG squared) given evidence of varied associations between CGG repeat length and behavioral measures in the PM (20, 21, 33). Given the presence of two X chromosomes in females, in models that included CGG repeat length, AR and an interaction term (i.e., CGG × AR) were included as covariates.

## Results

### Characterization and Group Differences on Clinical-Behavioral and Cognitive Measures

#### BAP

##### MPAS

Among those with MPAS data in the PM group (*n* = 87), 54% of the sample was characterized as BAP (+). Thirty-two percent of the sample exhibited either social or rigid BAP personality features (*n*s = 28), and 10% of the sample (*n* = 9) displayed both social and rigid BAP personality features. Prior studies using the MPAS indicate ~10–15% of controls display any BAP personality features (27, 46).

##### Pragmatic Rating Scale

**Figure 1:**
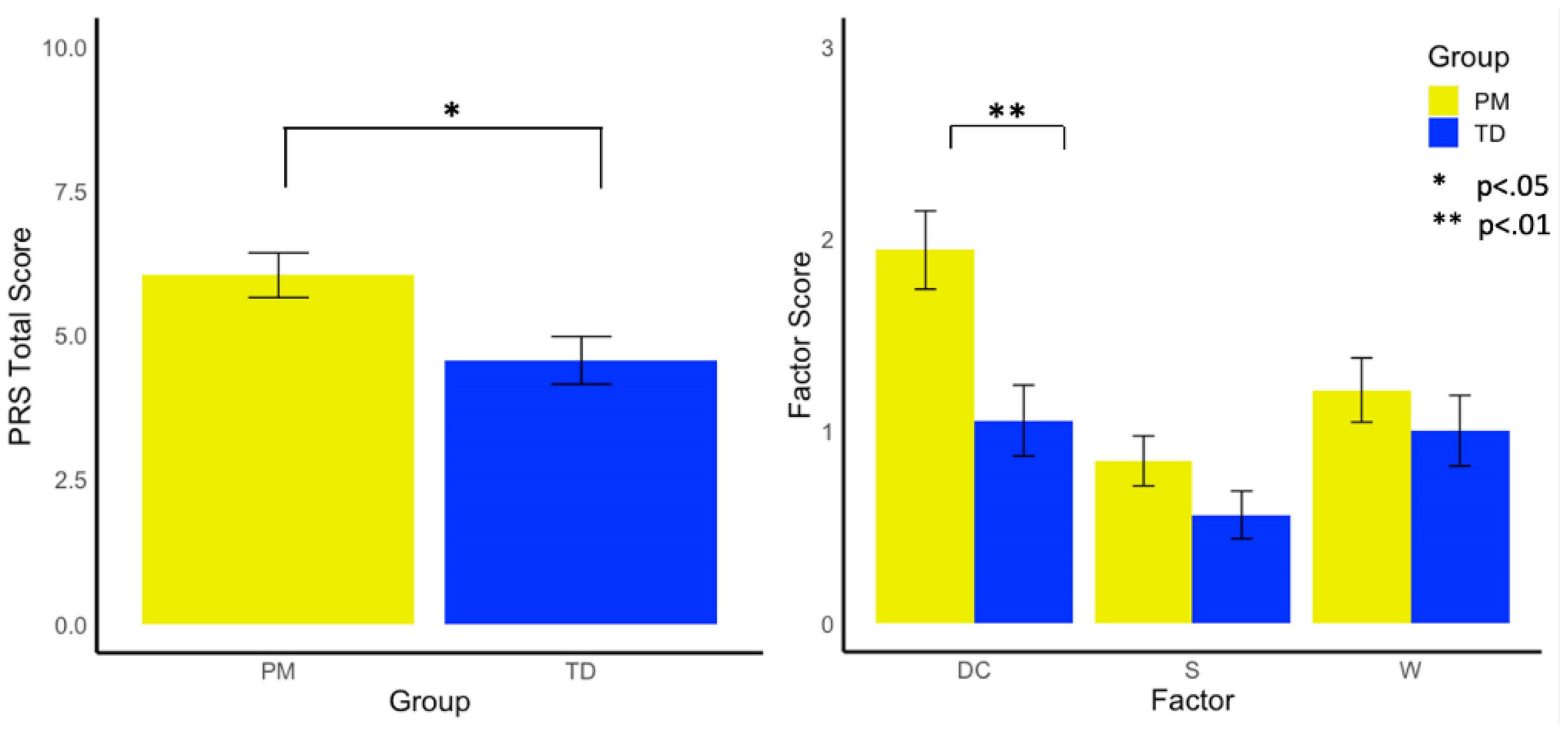
Group differences in pragmatic rating scale total scores and factors. DC, Dominates Conversation Factor; S, Suprasegmental Factor; W, Withdrawn Factor.

Females with the PM exhibited significantly higher total PRS scores (i.e., more violations) than the control group [*F*_(1, 185)_ = 5.65, *p* =0.019, and $\eta_{\text{p}}^{2}$ = 0.03]. Examining factor scores on the PRS revealed that the PM group demonstrated a more dominant conversational style than controls [*F*_(1,174)_ = 7.67, *p* =0.006, and $\eta_{\text{p}}^{2}$ = 0.04], but did not differ from controls in their withdrawn [*F*_(1,174)_ = 0.60, *p* = 0.439, and $\eta_{\text{p}}^{2}$ = 0.00] or suprasegmental scores [*F*_(1,174)_ = 2.08, *p* = 0.151, $\eta_{\text{p}}^{2}$ = 0.01, see Figure 1].

#### Mood and Anxiety

##### Beck Depression Inventory

PM carriers did not significantly differ from controls on BDI-II total scores [*F*_(1, 69)_ = 2.58, *p* = 0.113, $\eta_{\text{p}}^{2}$ = 0.04].

##### State Trait Anxiety Inventory

PM carriers endorsed marginally more Trait and State anxiety than controls [*F*_(1 77)_ = 3.72, *p* = 0.058, $\eta_{\text{p}}^{2}$ = 0.05; *F*_(1, 76)_ = 3.02, *p* = 0.086, $\eta_{\text{p}}^{2}$ = 0.04, respectively]. Clinically significant symptoms of anxiety (>39) were self-reported in 94.7% of PM carriers and 90% of controls.

##### MINI

PM carriers and controls did not differ on rates of current generalized anxiety disorder [22 vs. 14%, $X_{\left(1,144\right)}^{2}$ = 1.40, *p* = 0.285], past generalized anxiety disorder [19.6 vs. 11.9%, $X_{\left(1,144\right)}^{2}$ = 1.23, *p* = 0.338], current major depressive disorder [5 vs. 0%, $X_{\left(1,144\right)}^{2}$ = 2.75, *p* = 0.164], or past major depressive disorder [25.7 vs. 19.6%, $X_{\left(1,144\right)}^{2}$ = 0.75, *p* = 0.440].

#### Social Cognition

##### Reading the Mind in the Eyes

PM carriers had marginally lower scores on the Eyes Task than controls [*F*_(1, 203)_ = 1.49 *p* = 0.075, $\eta_{\text{p}}^{2}$ = 0.02].

##### Movie Stills

PM carriers demonstrated significantly lower overall performance on movie stills with faces [*F*_(1, 23)_ = 6.03, *p* = 0.015, $\eta_{\text{p}}^{2}$ = 0.03], but did not differ from controls in performance on stimuli without faces (*p* = 0.389). PM carriers had marginally lower scores on neutral and sad stimuli with faces (*p* = 0.074 and 0.069, respectively).

##### Point Light Basic

PM carriers scored significantly lower than controls on happy stimuli [*F*_(1, 97)_ = 4.25, *p* = 0.042, $\eta_{\text{p}}^{2}$ = 0.04], but did not differ in overall performance (*p* = 0.256) or on other emotion types (*p* > 0.145).

##### Point Light Trustworthiness

No differences emerged between groups in overall point light trustworthiness scores [*F*_(1, 24)_ = 0.94, *p* = 0.335, $\eta_{\text{p}}^{2}$ = 0.008], or for more trustworthy or less trustworthy stimuli [*F*_(1, 24)_ = 0.74, *p* = 0.392, $\eta_{\text{p}}^{2}$ = 0.006; *F*_(1, 24)_ = 0.65, *p* = 0.422, $\eta_{\text{p}}^{2}$ = 0.005, respectively].

##### Trustworthiness of Faces

**Figure 2:**
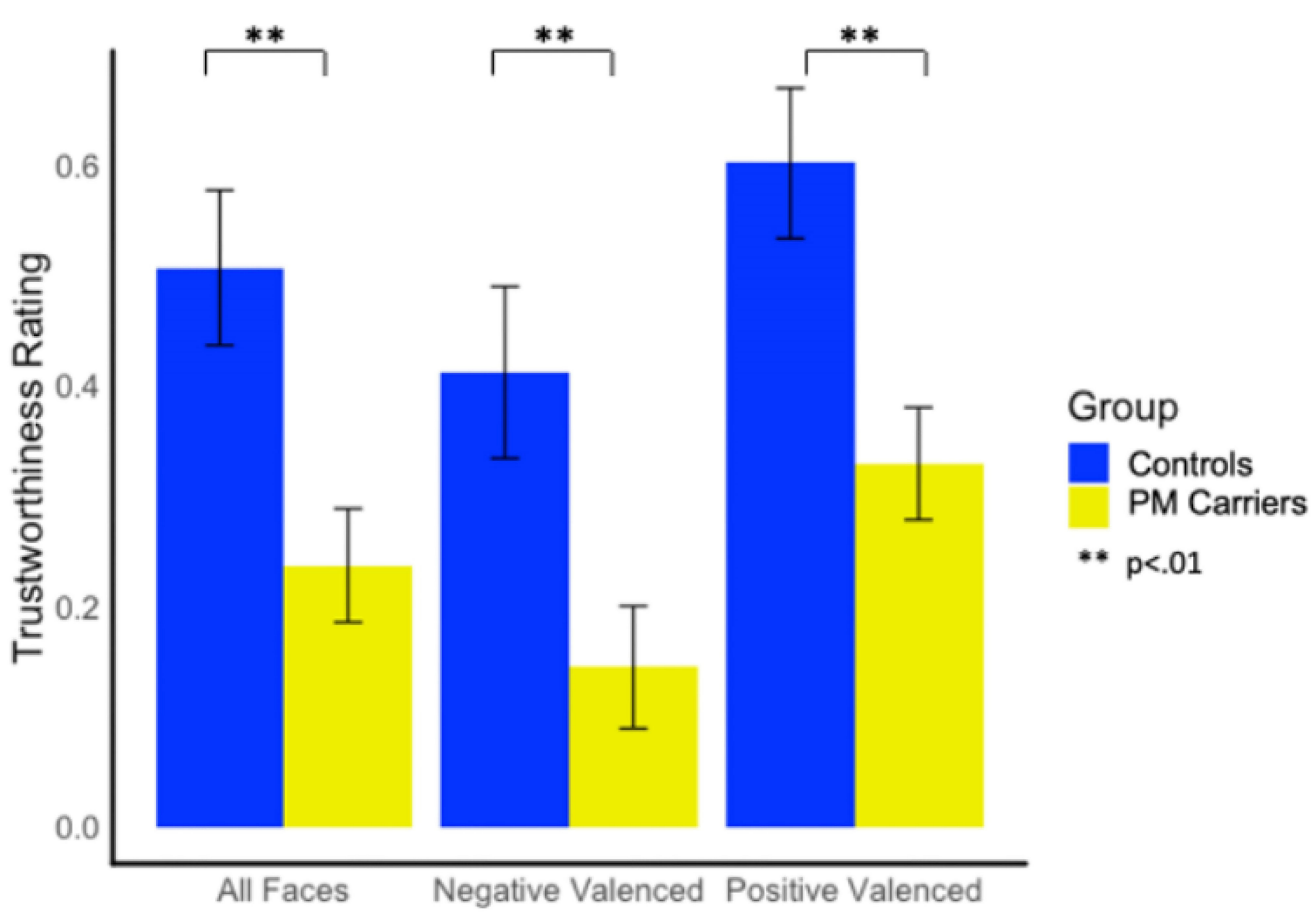
Group differences in trustworthiness ratings.

PM carriers rated faces as significantly less trustworthy than controls overall [*F*_(1, 147)_ = 9.31, *p* = 0.003, $\eta_{\text{p}}^{2}$ = 0.06]; differences were evident on both positive and negative valanced faces [*F*_(1, 147)_ = 9.80, *p* = 0.002, $\eta_{\text{p}}^{2}$ = 0.06; *F*_(1, 147)_ = 7.74, *p* = 0.006, $\eta_{\text{p}}^{2}$ = 0.05, respectively; see Figure 2].

#### Executive Function

##### BRIEF-A

**Figure 3:**
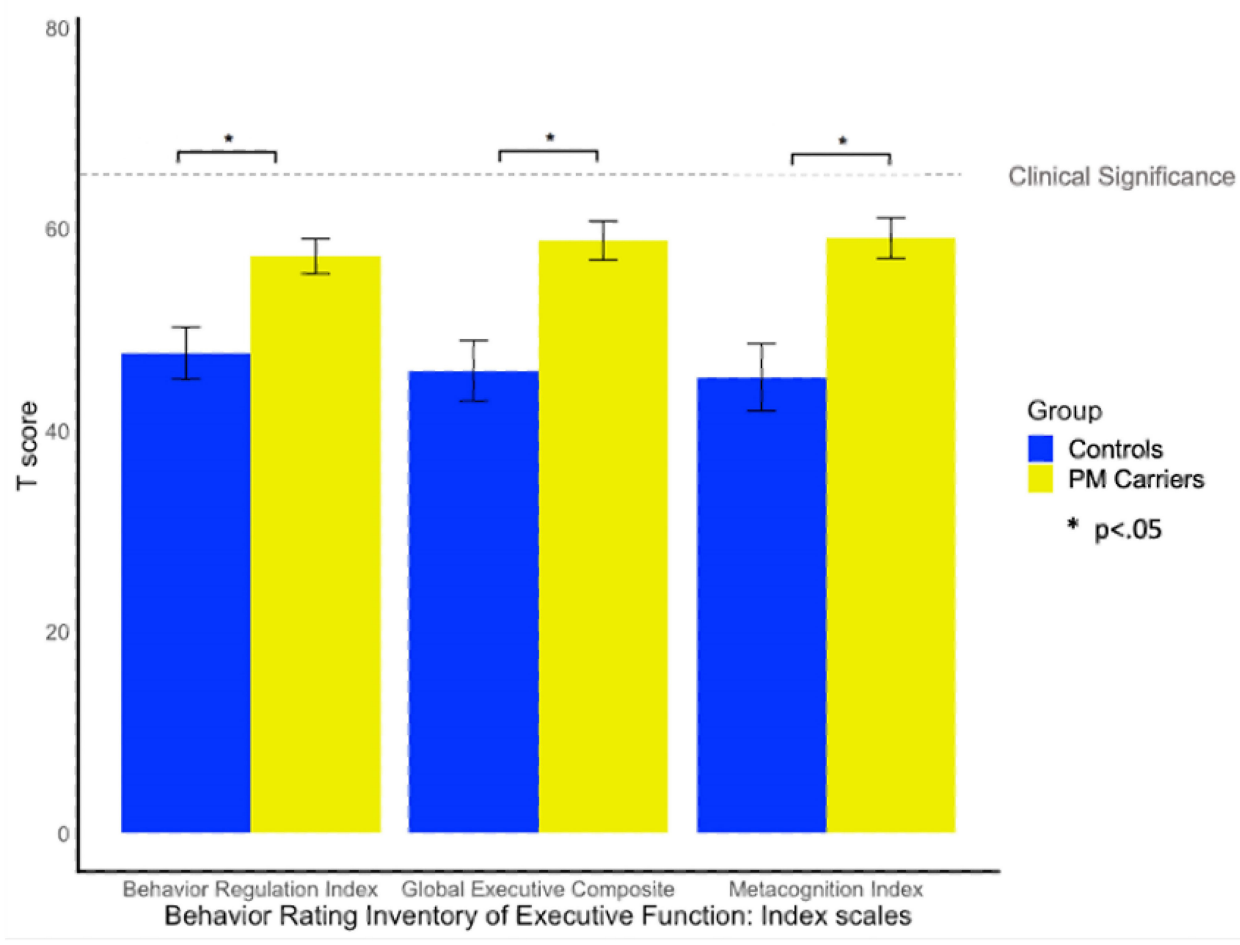
Group differences on the BRIEF-A. BRIEF-A *T*-scores > 65 indicate clinically elevated difficulties relative to the standardization sample.

Twenty-nine percent of PM carriers exceeded clinical cut-off on the GEC of the BRIEF-A, whereas only one female control exceeded clinical cut-off. PM carriers scored significantly higher (i.e., greater EF difficulty) than controls on the GEC, BRI, and MI scales [*F*_(1, 58)_ = 6.55, *p* = 0.013, $\eta_{\text{p}}^{2}$ = 0.10; *F*_(1, 58)_ = 4.47, *p* =0.039, $\eta_{\text{p}}^{2}$ = 0.07; *F*_(1, 58)_ = 6.81, *p* = 0.012, $\eta_{\text{p}}^{2}$ = 0.12; see Figure 3].

### Latent Profile Analysis of PM Carriers

LPA solutions for up to six models are presented in Table 4, and a three-profile solution was selected for interpretability.

**Table 4:** Indices of model fit for latent profile analysis.

**Number of profiles**	**AIC**	**BIC**	**aBIC**	**Entropy**	**BLRT *p*-value**	**Log likelihood**	**TRd**
1	3,782.24	3,866.90	3,778.29	–	–	−1,863.118	–
2	3,683.82	3,813.85	3,677.75	0.95	<0.001	−1,798.911	$\chi_{\left(15\right)}^{2}$ = 111.79, *p* < 0.001
**3**	**3,621.14**	**3,796.52**	**3,612.95**	**0.91**	**<0.001**	**−1,752.569**	$\chi_{\left(15\right)}^{2}$**= 99.07**, ***p*****< 0.001**
4	3,573.16	3,793.90	3,562.86	0.76	<0.001	−1,713.578	$\chi_{\left(15\right)}^{2}$ = 61.46, *p* < 0.001
5	3,546.71	3,812.82	3,534.30	0.78	<0.001	−1,685.357	$\chi_{\left(15\right)}^{2}$ = 54.79, *p* < 0.001
6	3,520.57	3,832.03	3,506.03	0.79	<0.001	−1,657.284	$\chi_{\left(15\right)}^{2}$ = 56.66, *p* < 0.001

*AIC, Akaike Information Criterion; BIC, Bayesian Information Criterion; aBIC, Adjusted Bayesian Criterion; BLRT, Bootstrap Likelihood Ratio Test; TRd, Sattora–Bentler Scaled Likelihood Ratio Chi-square Difference Test. The 3-class solution (bolded) was selected for final inclusion*.

**Figure 4:**
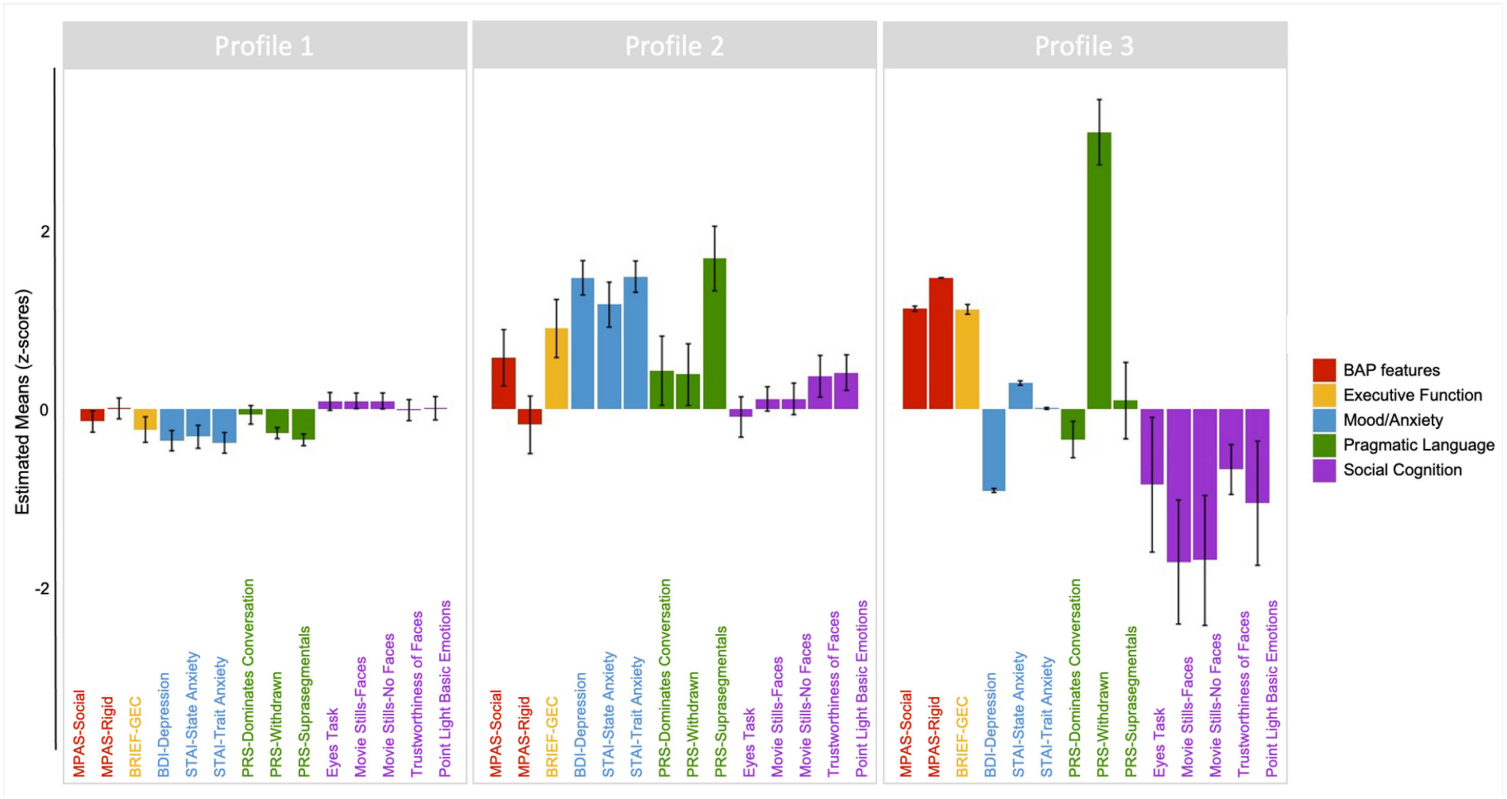
Latent profile groups emerging from performance profiles across clinical-behavioral, pragmatic language, social cognitive, and executive tasks. Positive scores reflect poorer or more atypical performance along the following: BAP personality features (red); Executive function (yellow); Mood/anxiety (blue); Pragmatic language features of the BAP (green). For social cognition (purple), negative scores reflect poorer performance relative to the mean.

The results of the three-profile solution are presented in Figure 4. The first profile (Profile 1) contained 77.6% of PM carriers (*n* = 118, average posterior probability = 0.983), and included individuals whose scores across domains consistently fell around the PM group mean, with limited variation across domains. The second profile (Profile 2) contained 17% of PM carriers (*n* = 26, average posterior probability = 0.871) and reflected individuals with increased mood and anxiety symptoms, slightly elevated social and rigid personality features of the BAP, and increased pragmatic language violations in the suprasegmental domain (e.g., intonation of voice, rate of speech, and volume modulation) as compared to other PM carriers. The final profile (Profile 3) contained 5.3% of PM carriers (*n* = 8, average posterior probability = 0.959) and included individuals who demonstrated elevated executive dysfunction, poorer social-cognitive abilities across tasks, elevated social and rigid personality features of the BAP, and increased pragmatic language violations in the listener expectation domain (e.g., unable to clarify, failure to reciprocate) relative to other PM carriers.

Follow up comparisons showed no significant differences across the profile groups in age, IQ, *FMR1*-related genetic variation, maternal status, or presence of ASD diagnosis or severity of ASD symptoms in their children (all *p*s > 0.200).

### Multiple Regressions: PM Phenotypes and *FMR1*-Related Variation

A linear regression model including CGG repeat length predicted a significant amount of variance in PRS scores (*R*^2^ = 0.09, *p* = 0.046; see Table 5). No other associations were observed between CGG repeat length and other clinical-behavioral, social-cognitive, or EF measures (*p* > 0.169).

**Table 5:** CGG repeat length predicts elevated pragmatic language violations (on Pragmatic Rating Scale).

	**Unstandardized *b* coefficient**	**S.E**.	**β**	***t***	***p*-value**
**CGG repeat length**	**−0.21**	**0.10**	**−0.96**	**−2.05**	**0.046**
Activation Ratio	−25.43	13.24	−1.70	−1.92	0.062
CGG*Activation Ratio	0.30	0.15	2.12	1.96	0.057

*All significant effects (p < 0.05) are noted in bold. CGG, Cytosine-Guanine-Guanine*.

FMRP was a significant predictor of performance on one social-cognitive task: Movie Stills (Happy) with faces. Specifically, quantitative FMRP predicted 12% of the variance in Movie Stills (Happy) with faces (*b* = −0.06, *p* = 0.005). Quantitative FMRP levels were not significantly associated with other clinical-behavioral, social-cognitive, or EF measures (*p* > 0.131).

## Discussion

This study characterized clinical-behavioral, social-cognitive, and EF features in females with the *FMR1* PM. Consistent with prior work, results revealed elevated rates of subclinical ASD-related personality and language features among women with the PM, as well as increased self-reported difficulties in executive functioning, but no differences in mood and anxiety symptoms compared to controls. Some differences in social-cognitive tasks were also observed, including differences in complex social-emotional judgements of trustworthiness of faces, and in accuracy identifying basic emotions when viewing complex scenes. Latent profile analysis revealed three subgroups within the PM group who exhibited distinct phenotypic profiles across clinical-behavioral, social-cognitive, and EF measures, which together with group differences, and some associations with *FMR1*-related variation, may provide insights into phenotypic profiles associated with the *FMR1* PM.

Replicating prior work, personality and pragmatic language differences that define the BAP were observed at elevated rates in the PM carrier group. Direct measurement tools, scored by raters blind to group status, identified approximately half of the PM carrier group as displaying personality traits consistent with the BAP, compared with published rates among individuals without a family history of ASD or FXS ranging from ~5 to 10% [e.g., (46, 80)]. Pragmatic language differences have been repeatedly observed among PM carriers (27, 57, 100–102), and were evident in this study as well, with additional patterns noted across the types of pragmatic language violations occurring more frequently in the PM group, who tended to display a more dominant conversational style (e.g., overly detailed, tangential language) than controls. Differences in pragmatic language have consistently emerged as a phenotypic marker associated with the PM in females (27, 100–102), and among individuals with FXS, particularly those who meet criteria for ASD (62, 103–109) and may be of clinical importance. For instance, prior work has shown that pragmatic language violations among mothers of children with FXS were associated with reduced self-reported quality of life for mothers (102), as well as elevated ASD symptoms and weaker expressive and receptive language in their children (27, 100). Together, this suggests that pragmatic language may be relevant to the well-being of both mothers and their children.

In contrast to previous work demonstrating higher rates of mood and anxiety disorders in PM carriers (25, 28, 30), we found no significant differences in depression scores between PM carriers and controls and only marginal differences from controls for anxiety symptoms, with both groups self-reporting elevated anxiety symptoms (>90% of participants). Rates of anxiety and depression were far lower in both groups using the MINI, a standardized psychiatric interview (ranging from 0 to 25%), and also comparable to rates reported in prior work (31, 33, 110). In follow-up analyses, no differences were observed on depression and anxiety features according to maternal status, or the number of affected children in each family, suggesting these findings are not related to parenting factors.

Unique to the present study was the inclusion of multiple measures of social cognition within a single sample, permitting characterization of social cognitive strengths and weaknesses across different types of stimuli. Differences in social-cognitive profiles have been reported among PM carriers compared to controls, particularly among males (35, 37). This study identified key social-cognitive differences in female carriers of the PM in specific tasks, which echo findings observed among individuals with FXS (62, 111). PM carriers differed in rating trustworthiness in response to faces ranging in emotional valence, a task that draws on social-cognitive and social decision-making skills. Differences on this task have been reported in ASD and the BAP in clinically unaffected relatives (61), and also among patients with bilateral amygdala damage, implicating this brain region in atypical performance in these groups (87, 89, 112). In studies of the PM, differences in amygdala volume and activation have been linked to aspects of social cognition among male carriers (35, 37), and these findings may suggest similar relationships among women with the PM that will be important to investigate in future work.

PM carriers were also less accurate in inferring emotions from faces when viewing still movie scenes. These results may indicate that PM carriers use different strategies in making social judgments relative to controls when viewing faces. Prior work that used eye tracking to examine looking patterns in response to faces indicates that PM carriers use different visual strategies from controls to inform social judgments (63). In the present study, marginal differences were also observed on the task involving reading complex thoughts and emotions from the eye region of the face. Thus, sensitivity to gaze and emotion expression, as measured by these social-cognitive tasks, may be an objective behavioral marker of underlying neural processes associated with interpreting emotion within the PM (35).

Consistent with prior reports of EF impairment associated with the PM (113), the PM group exhibited significantly higher executive functioning difficulty than controls, with approximately one third of the PM group reporting clinically significant EF difficulties. Characterization of the cognitive phenotype associated with the PM is especially important for understanding the manifestation of the neurodegenerative disorder, FXTAS, which may reveal subclinical phenotypic markers that are evident in a subgroup of PM carriers who go on to develop the disorder, as dysexecutive symptoms are a hallmark of FXTAS, particularly in males (114). Nonetheless, females with the PM exhibit differences in EF symptoms even without a diagnosis of FXTAS (68, 69, 115, 116). This study highlights the value of using self-report measures of EF in studies of the PM, as prior work has typically used online behavioral measures [e.g., see (113) for review]. The use of the BRIEF-A among females with the PM has been limited to only a handful of prior studies (23, 117), but is useful in that clinical significance may be easily determined and a standardized self-report measure can enable cross-cohort comparisons. Of note, however, such self-report measures could lead to over-reporting of symptoms (118, 119), and so may best be interpreted within the context of results from studies employing direct assessment of executive skills.

Complementing results from group comparisons, latent profile analyses revealed three distinct groups of PM carriers who displayed different profiles of performance across the various domains. Profile 1 comprised the largest subgroup, and represented those scoring at the mean of the sample across measures, and who largely contributed to the group differences observed in pragmatic language and select social cognitive tasks and EF. Profile 2 included 17% of the sample, and was characterized by elevated and co-occurring mood and anxiety symptoms, mild expression of personality features of the BAP, and higher suprasegmental violations (e.g., atypical variation in intonation, volume, or rate of speech). This specific co-occurrence of features is not surprising in the context of social-emotional patterns commonly observed among individuals who are more anxious or depressed, in that their symptoms can interfere with their social relationships, and vice versa (120). Allen et al. (55) also reported distinct clusters of PM carriers who reported different mood and anxiety symptoms, and other work has reported the co-occurrence of mood/anxiety features with executive dysfunction among PM carriers (54). Consistent with prior work (121), this subgroup also exhibited slightly elevated BAP traits, which raises the possibility that the co-occurrence of such traits may also be common to a subgroup of family members of individuals with ASD (of note, individuals showing Profile 3, discussed below, demonstrated elevated BAP features in the absence of elevated mood/anxiety symptoms). Although suprasegmental speech violations committed by PM carriers in this subgroup does not constitute clinical impairment, among individuals with ASD, where suprasegmental atypicalities are more pronounced, such variation can pose a significant obstacle to social interactions (122). First-degree relatives of individuals with ASD also demonstrate subtle differences in suprasegmental aspects of language (e.g., prosody) (123, 124).

Profile 3 represented the smallest subgroup, comprised of ~5% of PM participants who exhibited starker differences across all clinical-behavioral, social-cognitive, and executive domains relative to other PM carriers. This subgroup displayed notable differences on social-cognitive tasks together with elevated BAP features, and high rates of pragmatic language violations in particular. This pattern of performance is markedly similar to features described in prior investigations of parents of individuals with ASD who display the BAP (60, 61). Given the large number of ASD risk genes known to interact with *FMR1* (50, 51), it may be that this phenotypic profile reflects an increased genetic liability for ASD among this subgroup. However, the small size of Profile 3 warrants cautious interpretation. We found no group differences in age, IQ, or *FMR1*-related variation between the Profile subgroups, likely due in part to some missing data in Profile 3 and unequal sample sizes across groups, and further investigations in larger samples will be important for confirming these patterns. Importantly, membership within any of the profile groups was not associated with differences in age, IQ, or parenting stress-related factors (including number of affected children, or severity of child symptoms), suggesting the phenotypic profiles identified are likely reflective of inherent traits, rather than systematic environmental differences between subgroups.

Finally, some associations were detected between phenotypic profiles and *FMR1*-related molecular genetic variability in the PM carrier group. We found linear associations between CGG repeat length and pragmatic language, indicating greater violations at *lower* ends of the CGG continuum. The CGG range reflected in the participants studied extended from 59 to 126 CGGs; thus, it may be that inclusion of more PM carriers with higher repeats (i.e., over 120) might have altered the findings observed here. Prior work has observed curvilinear links between language and CGG length, with different patterns noted in the mid-range (90–110 repeats) as compared to those with CGG repeats beyond 120 (21). Interestingly, the linear CGG association became marginal after factoring in participants' activation ratios, suggesting the importance of considering the second, healthy X allele in phenotype-genotype associations of females with the *FMR1* PM. Additionally, we found that higher levels of FMRP predicted poorer performance on one social-cognitive task. This finding is somewhat in contrast to those from Hessl et al. (37), who found associations between *reduced* FMRP and poorer performance on social processing tasks in male PM carriers. Interestingly, a prior study based on the PM sample of participants studied here reported increased FMRP related to poorer performance on a language fluency task that taps into executive functioning (22), which together may suggest that the findings observed here may be specific to our sample of PM carriers (and thus may not be replicable), or that FMRP from blood is not analogous to FMRP in the brain. The links between *FMR1* and the phenotypes included in the present study are likely not straightforward, and it is possible that other *FMR1*-related factors (e.g., mosaicism, mRNA) could also help to elucidate *FMR1*-associated patterns not explored in this study (125, 126).

### Strengths, Limitations, and Future Directions

Together, findings contribute to an emerging profile of PM carriers that suggests substantial phenotypic variability, and the presence of distinct phenotypic subgroups that may reveal important differences in underlying mechanistic factors and etiology (e.g., involvement of ASD risk genes interacting with *FMR1*). Strengths of this study included the broad phenotypic characterization of a relatively large group of females with the PM. Examining an array of phenotypic measures in a single sample enabled us to investigate comparisons with controls as well as unique profiles among PM carriers. Nevertheless, a larger sample may have mitigated some of the concerns with the small subgroup sample sizes yielded in our latent profile analyses. We were limited in our attempts at a validation analysis of the three-profile model through comparison across such important factors as *FMR1*-related variation, though it is possible that other individual factors not available in the present study might have differentiated subgroups, such as direct measures of caregiving stress (127, 128), the presence of other co-occurring health conditions (55), or polygenic risk for ASD (47). There is emerging literature to suggest subgroups among PM carriers, and indeed the clinical disorders associated with the PM (i.e., FXTAS, FXPOI, and FXAND) only occur among a subset of PM carriers [e.g., see (55), and for review, (113, 129)]. Large scale studies of the PM are warranted to further investigate the interrelationships observed here, and to determine whether the phenotypes included in this study may co-occur with other meaningful clinical or health outcomes, as has been documented previously (55). Additionally, we were limited in *FMR1*-related information, as we did not have genetic data on the control group in this study. Further, there is emerging evidence to suggest that phenotypic associations may be observed across the range of CGG repeats (21, 130, 131). It may be that *FMR1* relationships with phenotypes were not detected given that the range of repeats in the current study was limited to the PM range. It may also be that molecular parameters in blood do not correlate with cognitive assays in the brain in straightforward ways. We recognize that due to our exclusion criteria, our control group might be expected to have performed well on the measures employed here. Larger and more heterogeneous control groups should be included in future studies, or in comparison to mothers of children with ASD. Finally, it will be important for future work to examine whether the findings reported here may extend to males with the PM, given prior evidence to suggest females and males with the PM may exhibit somewhat different phenotypic profiles and associations with underlying biology (29, 132). Such studies may build on the present findings, and help to characterize the phenotypic profile associated with the *FMR1* PM, and inform clinical efforts to promote the health and well-being of individuals with the PM and their families.

## Conclusions

This study provided a comprehensive assessment of clinical and subclinical phenotypes associated with the *FMR1* PM. We identified differences from controls on pragmatic language features of the BAP, executive functioning, and some aspects of social cognition, but did not observe differences in mood and anxiety. Using LPA, we found subgroups within the PM sample characterized by unique patterns of performance on these measures. This study adds to a growing literature suggestive of important phenotypic heterogeneity among PM carriers, and provides further insight into *FMR1*-associated phenotypes.

## Data Availability Statement

The datasets presented in this study can be found in online repositories. The names of the repository/repositories and accession number(s) can be found below: https://nda.nih.gov/edit_collection.html?id=1958.

## Ethics Statement

The studies involving human participants were reviewed and approved by Northwestern University Institutional Review Board. The patients/participants provided their written informed consent to participate in this study.

## Author Contributions

NM helped to conceptualize the project, drafted the initial manuscript, and analyzed the data. JG, KN, MW, and JL assisted in preparing the dataset and provided valuable feedback on manuscript drafts. GM contributed to manuscript preparation. LB, SP, and MLe contributed to data preparation and processing. JS provided further consultation and verification of statistical methods and results. DH advised on PM participant evaluation of neurocognitive functioning. LZ, KS, and EB-K provided resources for genetic analysis of the FMR1 gene, helped to draft the Materials and Methods section pertaining to genetic analyses, and helped with interpretation of results. MLo secured funding for the projects from which these data were drawn, conceptualized the project, helped to interpret data, and develop the manuscript. All authors reviewed and approved the manuscript.

## Conflict of Interest

The authors declare that the research was conducted in the absence of any commercial or financial relationships that could be construed as a potential conflict of interest.

## Publisher's Note

All claims expressed in this article are solely those of the authors and do not necessarily represent those of their affiliated organizations, or those of the publisher, the editors and the reviewers. Any product that may be evaluated in this article, or claim that may be made by its manufacturer, is not guaranteed or endorsed by the publisher.
