# Supplementary material for: The Phenotypic Profile Associated With the FMR1 Premutation in Women: An Investigation of Clinical-Behavioral, Social-Cognitive, and Executive Abilities
Source: Front Psychiatry. 2021 Aug 6;12:718485. doi: 10.3389/fpsyt.2021.718485 (PMC8377357; doi:10.3389/fpsyt.2021.718485)
Supplement: Supplementary file 1 [file Data_Sheet_1.DOCX]

**Supplementary Materials**

**Latent profile analysis**

Latent profile analysis (LPA) was performed using the robust maximum likelihood (MLR) estimator with Mplus: Sixth Edition (Muthén and Muthén 2007). Data missingness (39.85% total across all variables from the PM group) was assessed and determined to be random, and as such, full information maximum likelihood estimation (FIML) was utilized to impute the data (Vink et al., 2014). Given numerical scale differences between measures, all variables were *z*-scored to improve interpretability.

LPA models yielding one- to six-profile solutions were conducted iteratively. From these solutions, metrics of Akaike Information Criterion (AIC), the Bayesian Information Criterion (BIC), Entropy, a Bootstrap Likelihood Ratio Test (BLRT), and a Sattora-Bentler Scaled likelihood ratio chi-square difference test (TRd) were computed to evaluate changes to model fit as the number of latent profiles increased (Tein et al., 2013;Williams and Kibowski, 2016;Ferguson et al., 2019;Spurk et al., 2020). AIC and BIC are commonly used measures of model selection, and lower values, relative to the previous model, indicate better model fit. Entropy values range from 0-1, with values closer to 1 indicating greater fit and values >.80 suggest that individuals are being appropriately assigned to groups (Tein et al., 2013). The BLRT and TRd are used to determine whether there is a significant improvement in the model fit with the inclusion of additional profiles. Posterior probability for each profile is included, with values exceeding .70 indicative of certainty with which group membership is assigned that exceed those assigned by chance (Ram and Grimm, 2009;Berlin et al., 2014). In addition to statistical metrics for model fit, we considered the theoretical interpretability of the profiles and profile size. Notably, however, given the heterogeneity among PM carriers, it would not be unexpected for a small subgroup to emerge in this population. As such, model solutions producing small profile groups were considered in combination with metrics of model fit.

Fit statistics and theoretical meaning of the profiles were considered when determining which model best fit the data. A 3-profile model was selected for interpretability, which had low AIC/BIC relative to other solutions and high entropy (>.90). While chi-square tests indicated that model fit improved incrementally from 4- to 6- profile solutions, this was at the expense of entropy, and examination of the patterns of performance in 4- to 6- profile models suggested that the additional profiles did not substantively discriminate between profile membership.

To evaluate whether there were any meaningful differences across the latent profiles identified within the PM group on the individual factors of age, IQ, *FMR1*-related genetic variation, and severity of autism symptoms in their children, nonparametric tests were performed, so as to account for unbalanced sample sizes based on profile membership. For evaluations of group differences in age, IQ, *FMR1*, and ADOS-2 scores, separate Kruskal-Wallis one-way ANOVAs were used when evaluating LPA solutions with more than two profiles. In the case of a small subgroup associated with a particular profile membership or insufficient data for a particular measure within a subgroup (*n*<5), Wilcoxon ranked-sum tests were used in place of the ANOVAs to evaluate the differences between two profiles only. A chi-square test was used to examine the association between profile membership and their child’s ASD status.

**References**

Berlin, K.S., Williams, N.A., and Parra, G.R. (2014). An introduction to latent variable mixture modeling (part 1): overview and cross-sectional latent class and latent profile analyses. *J Pediatr Psychol* 39**,** 174-187.

Ferguson, S.L., Whitney, E., Moore, G., and Hull, D. (2019). Finding latent groups in observed data: A primer on latent profile analysis in Mplus for applied researchers. *International Journal of Behavioral Development* 44**,** 458-468.

Losh, M., and Piven, J. (2007). Social-cognition and the broad autism phenotype: identifying genetically meaningful phenotypes. *J Child Psychol Psychiatry* 48**,** 105-112.

Mervis, C.B., and Klein-Tasman, B.P. (2004). Methodological issues in group-matching designs: alpha levels for control variable comparisons and measurement characteristics of control and target variables. *J Autism Dev Disord* 34**,** 7-17.

Muthén , L.K., and Muthén , B.O. (2007). "Mplus Users Guide". (Los Angeles, CA: Muthén & Muthén).

Ram, N., and Grimm, K.J. (2009). Growth Mixture Modeling: A Method for Identifying Differences in Longitudinal Change Among Unobserved Groups. *Int J Behav Dev* 33**,** 565-576.

Spurk, D., Hirschi, A., Wang, M., Valero, D., and Kauffeld, S. (2020). Latent profile analysis: A review and "how to" guide of its application within vocational behavior research. *Journal of Vocational Behavior* 120.

Tein, J.Y., Coxe, S., and Cham, H. (2013). Statistical Power to Detect the Correct Number of Classes in Latent Profile Analysis. *Struct Equ Modeling* 20**,** 640-657.

Vink, G., Frank, L.E., Pannekoek, J., and Van Buuren, S. (2014). Predictive mean matching imputation of semicontinuous variables. *Statistica Neerlandica* 68**,** 61-90.

Williams, G.A., and Kibowski, F. (2016). "Latent class analysis and latent profile analysis," in *Handbook of Methodological Approaches to Community-Based Research: Qualitative, Quantitative, and Mixed Methods*. (New York: Oxford University Press), 143-151.
